# Fragment-Derived Nicotinic Acid Analogues Inhibit hCA III and Downregulate *CA3* Expression in HepG2 Cells

**DOI:** 10.3390/biom16040599

**Published:** 2026-04-17

**Authors:** Areej Abuhammad, Tamara Sabri, Nidaa A. Ababneh, Rya A. Ali, Mohammad A. Ismail, Adan Madadha, Dareen T. Yazjeen, Rama J. Alghanem, Ali M. Qaisi, Yusuf Al-Hiari, Kapil Gupta, Imre Berger, Edith Sim

**Affiliations:** 1 Department of Pharmaceutical Sciences, School of Pharmacy, The University of Jordan, Amman 11942, Jordan; 2 Cell Therapy Center, The University of Jordan, Amman 11942, Jordan; 3 School of Biochemistry, University of Bristol, Bristol BS8 1TD, UK; 4 Bristol Synthetic Biology Centre BrisSynBio, Bristol BS8 1TD, UK; 5 Max Planck Bristol Centre for Minimal Biology, Bristol BS8 1TS, UK; 6 School of Chemistry, University of Bristol, Bristol BS8 1TS, UK; 7 Department of Pharmacology, University of Oxford, Oxford OX1 3QT, UK

**Keywords:** carbonic anhydrase III, fragment-based drug discovery, oxidative stress, nicotinic acid derivatives, metabolic disease, gene expression modulation

## Abstract

Chronic oxidative stress and lipid imbalance drive metabolic disorders such as obesity and non-alcoholic fatty liver disease, yet few therapies target the upstream redox imbalance in key tissues. Human carbonic anhydrase III (hCA III), a redox-associated enzyme enriched in liver and adipose tissue, has long remained pharmacologically elusive due to its low catalytic activity and lack of modulators. Here, we identify fragment-like nicotinic acid derivatives as non-sulfonamide hCA III modulators and evaluate their associated cellular effects. Using an esterase activity assay, we screened 25 analogues and identified two fragment-like hits, compound **17** (2-thioethyl) and compound **22** (6-morpholino), with IC_50_ values of 487 and 361 µM, respectively. Orthogonal thermal shift analysis supported compound-protein interaction, and selected hits were subsequently evaluated in HepG2 cells. Both compounds were associated with reduced *CA3* mRNA expression after treatment at 1 µM, while their cellular phenotypes diverged, with compound **22** increasing ROS under oxidative stress conditions and compound **17** affecting mitochondrial membrane potential. Taken together, these findings identify tractable nicotinic acid-derived fragment hits and associated cellular phenotypes that warrant further mechanistic investigation. These fragment-like hits provide a practical starting point for studying the redox-linked biology of hCA III.

## 1. Introduction

Modern metabolic disorders, including obesity, non-alcoholic fatty liver disease (NAFLD), and type 2 diabetes, now affect over one billion individuals worldwide, posing a major and growing challenge to global health systems and pharmaceutical innovation [1,2,3]. These complex diseases are closely interconnected and share key pathophysiological drivers, most notably the chronic dysregulation of lipid metabolism and persistent oxidative stress [4,5]. In hepatocytes and adipose tissue, this redox imbalance triggers inflammation, insulin resistance, mitochondrial dysfunction, and progressive metabolic decline [6,7]. Most existing therapies fail to address these upstream molecular disruptions, highlighting the urgent need for targeted strategies that restore cellular homeostasis. One promising approach is to modulate redox-sensitive enzymes in liver and adipose tissue, as these tissues play central roles in oxidative stress responses and metabolic homeostasis [8].

One such enzyme is human carbonic anhydrase III (hCA III), a zinc metalloenzyme selectively expressed in the liver, adipose tissue, and oxidative skeletal muscle [9,10]. Although hCA III belongs to the α-carbonic anhydrase family, it has exceptionally low catalytic activity despite retaining the conserved zinc-binding active site for CO_2_ hydration. Its activity is strongly inhibited by bicarbonate, the reaction product, and it shows markedly lower sensitivity to classical sulfonamide inhibitors such as acetazolamide compared with other isoforms [11,12,13]. This low anhydrase activity has shifted interest toward potential non-catalytic roles of hCA III. Several studies suggest roles in oxidative stress defence, apoptosis regulation, and nutrient-responsive signaling [14,15]. Furthermore, its expression is nutritionally and hormonally regulated, and knockout studies suggest a role in maintaining metabolic homeostasis independent of catalytic function [16,17,18,19].

These proposed non-catalytic roles are particularly compelling in the context of oxidative stress and metabolic disease, where hCA III may contribute to cellular defence through a mechanism independent of CO_2_ hydration. The enzyme carries two surface-exposed cysteine residues (Cys183 and Cys188) that undergo reversible S-glutathionylation under oxidative conditions, a modification that protects against irreversible protein oxidation without affecting catalytic activity [20,21]. This cysteine-based redox buffering is consistent with earlier work showing that *CA3* overexpression reduces intracellular reactive oxygen species and protects cells from hydrogen peroxide-induced apoptosis, whereas its silencing sensitizes cells to oxidative cell death [15,22]. In the liver specifically, *CA3* expression is upregulated during hepatic fat accumulation even prior to overt obesity, and pharmacological suppression of CA III activity reduces lipid accumulation in hepatocytes [17]. More recently, CA III was identified as a direct binding partner of squalene epoxidase (SQLE), with the SQLE/CA III axis driving de novo lipogenesis and NF-κB-mediated inflammation in NASH; combined pharmacological targeting of both proteins synergistically ameliorated disease in multiple mouse models [23]. *CA3* has further been shown to regulate adipogenesis through modulation of PPARγ2, linking its expression to fat cell differentiation and energy storage [24]. Taken together, these findings support hCA III as a pharmacologically relevant target in oxidative stress and metabolic disease and highlight the need for selective chemical tools to explore its tissue-specific and potentially non-catalytic functions.

Despite its biological relevance, hCA III remains a technically challenging target for small-molecule discovery. Its exceptionally low hydratase activity renders CO_2_ hydration-based assays unsuitable for high-throughput screening [25,26], while the absence of ligand-bound crystal structures hinders structure-guided design. In addition, most available tools focus on catalytic inhibition and overlook redox-modulatory or non-enzymatic functions of CA III. Methods to study conformational changes or non-catalytic ligand binding in hCA III are still limited. Notably, prior mechanistic work on bovine CA III showed that hydrolysis of 4-nitrophenyl acetate can occur at a site distinct from that responsible for CO_2_ hydration, in contrast to CA I and CA II, in which the two activities are more closely linked [27]. In this context, the esterase assay is not only a practical surrogate for screening, but also a potentially informative readout of ligand engagement at a region of hCA III that may be relevant to its non-catalytic functions and that is amenable to adaptation for higher-throughput screening formats. At the same time, the physiological significance of carbonic anhydrase esterase activity remains unresolved, and its contribution to the proposed non-catalytic roles of hCA III has yet to be defined. Consequently, although recent sulfonamide-based inhibitors have shown potent and isoform-selective activity against CA III [28], non-sulfonamide inhibitors are still largely unexplored, with no candidates advancing beyond early hit identification. Together, these challenges highlight the need for innovative screening strategies, such as fragment-based approaches coupled with complementary biochemical and cellular assays.

Carboxylic acids such as nicotinic acid are well-established metalloenzyme inhibitors that typically act by coordinating to the catalytic zinc ion or displacing zinc-bound water in their anionic form [29]. Several nicotinic acid analogues, including acipimox, have shown micromolar inhibition of various carbonic anhydrase isoforms, including hCA III [30]. Although carboxylate binding has been structurally characterised in several carbonic anhydrase isoforms, the extent to which nicotinic acid analogues can achieve isoform selectivity, particularly for hCA III, remains unclear. Nicotinic acid is also a small, fragment-like scaffold with favourable aqueous solubility and a compact size that supports cellular testing, while substitution on the ring can be used to tune polarity and overall physicochemical behaviour. Importantly, nicotinic acid has also been reported to exert antioxidant and cytoprotective effects in oxidative stress models, including hepatocyte injury settings [31,32], lending additional biological relevance to this chemotype in redox-linked cellular contexts. Computational and chromatographic studies using bovine CA III suggest that nicotinic acid derivatives may bind its active site [33], but no direct biochemical or cellular validation exists for the human enzyme. This gap presents an opportunity to systematically assess nicotinic acid-based compounds as fragment-like probes for hCA III.

To test this, we screened a focused series of ring-substituted nicotinic acid analogues against recombinant hCA III using an esterase-based assay that can be adapted to high-throughput screening in future work. Hit prioritisation was supported by dose–response analysis and an orthogonal thermal shift assay to confirm ligand engagement. Selected compounds were then evaluated in a hepatocyte model (HepG2) to determine whether the prioritised hits were associated with measurable changes in *CA3* gene expression. Together, this combined biochemical, biophysical, and cellular approach provides a first step toward developing selective chemical tools to better understand the proposed redox-linked functions of hCA III.

## 2. Materials and Methods

### 2.1. Materials

Unless otherwise specified, all reagents and solvents were obtained from Sigma-Aldrich (Steinheim, Germany), Acros Organics (Geel, Belgium), or Fisher Scientific (Loughborough, UK), and were of analytical grade or higher. Nicotinic acid derivatives (compounds **2**–**26**) were purchased as commercially available compounds with reported purities of ≥98% and used without further purification. The parent compound, nicotinic acid, was obtained at ≥99% purity. Stock solutions of all compounds were prepared in dimethyl sulfoxide (DMSO; ≥99.9%, molecular biology grade).

Esterase activity assays were performed using molecular biology-grade reagents including 4-nitrophenyl acetate (4-NPA), Tris base, and sodium chloride, all sourced from Sigma-Aldrich (Steinheim, Germany). Quercetin (≥95%, HPLC grade) was used as a positive control in inhibition assays [34].

Recombinant expression of hCA III was carried out in *Escherichia coli* BL21(DE3) cells (Promega, Madison, WI, USA) cultured in 2 × YT medium (Fisher Scientific, Loughborough, UK), with protein induction triggered by isopropyl β-D-1-thiogalactopyranoside (IPTG). Restriction enzymes and cDNA synthesis kits used for *CA3* cloning were sourced from New England Biolabs (Hitchin, UK) and Promega (Madison, WI, USA).

HepG2 cells were cultured in Minimum Essential Medium (MEM, Euroclone, Milan, Italy) supplemented with fetal bovine serum (FBS, Cytiva, Little Chalfont, UK), GlutaMAX, and antibiotic-antimycotic solution (both from Gibco, Thermo Fisher Scientific, Paisley, UK). Corning^®^ half-area 96-well plates (Corning, Corning, NY, USA) were used for all assays: clear plates for absorbance-based measurements (e.g., esterase, MTT) and black plates for fluorescence-based assays (e.g., ROS, JC-1). Ultrapure type I water was generated in-house using a Smart2Pure^TM^ Water Purification System (Thermo Fisher Scientific, Waltham, MA, USA).

### 2.2. Expression and Purification of Recombinant hCA III

Recombinant hCA III was expressed using a codon-optimized construct (Gene ID: 761; Plasmid ID: HsCD00287898, PlasmID repository) cloned into the pACE expression vector, incorporating an N-terminal His_10_ tag removable by TEV protease cleavage. The plasmid was introduced into *E. coli* BL21(DE3) cells (Promega, Madison, WI, USA), and transformed colonies were grown overnight in 2 × YT broth supplemented with 100 µg/mL ampicillin at 37 °C.

For protein production, 500 mL cultures were inoculated at a 1:50 ratio and cultivated at 37 °C until reaching an optical density at 600 nm of approximately 1.0. Protein expression was induced with 0.1 mM IPTG, and cultures were incubated for 16 h at 18 °C. Cells were harvested by centrifugation at 4000× *g* for 20 min at 4 °C and stored at −80 °C.

Cell pellets were resuspended in lysis buffer (50 mM HEPES (pH 7.5), 500 mM NaCl, 5% glycerol, 0.5 mM TCEP, 5 mM imidazole, and an EDTA-free protease inhibitor cocktail), and disrupted via sonication. The lysate was clarified by centrifugation (15,000× *g*, 30 min, 4 °C), and the supernatant was applied to a nickel-charged affinity resin pre-equilibrated with lysis buffer. After extensive washing with buffer containing 40 mM imidazole, the bound protein was eluted using 250 mM imidazole.

Eluted fractions were pooled and dialyzed overnight at 4 °C into cleavage buffer (50 mM HEPES (pH 7.5), 150 mM NaCl, 0.5 mM TCEP, 5% glycerol), followed by TEV protease digestion to remove the His-tag. The cleaved protein was then concentrated and further purified using size-exclusion chromatography (SEC) on a Superdex 200 Increase 10/300 GL column (Cytiva, Little Chalfont, UK) equilibrated with storage buffer (20 mM HEPES (pH 7.5), 150 mM NaCl, 0.5 mM TCEP, 5% glycerol). Protein purity was assessed by SDS-PAGE (12%), which revealed a single band corresponding to the expected molecular weight of hCA III (~29 kDa, Figure S1, Supporting Information). Elution profiles confirmed the monomeric state and homogeneity of the purified protein (Figure S2, Supporting Information). The final protein was concentrated to ~10 mg/mL, aliquoted, flash-frozen in liquid nitrogen, and stored at −80 °C for downstream applications.

### 2.3. Esterase Activity Assay of hCA III

The enzymatic activity of recombinant hCA III was evaluated using a continuous colorimetric assay based on the hydrolysis of 4-NPA. The recombinant protein was prepared as described in Section 2.2. All assays were conducted in 96-well half-area microplates at 25 °C using a TECAN Infinite 200 PRO M Plex plate reader (TECAN, Grödig, Austria). Reactions were carried out in a total volume of 30 µL, with hCA III at a final concentration of 250 µg/mL in assay buffer composed of 20 mM Tris-HCl (pH 7.5) and 150 mM NaCl. The reaction was initiated by adding 4-NPA to a final concentration of 250 µM. Conditions were optimized to ensure a linear rate of product formation during the measurement period. Absorbance was recorded at 405 nm every 60 s for 60 min, and product concentrations were determined using a calibration curve generated with known concentrations of 4-nitrophenol (reaction product).

For inhibition studies, compounds were dissolved in DMSO and pre-incubated with the enzyme at room temperature (22 °C) for 30 min prior to substrate addition. The DMSO concentration was kept constant at 3.3% in all wells. Enzyme activity in the presence of inhibitors was normalised to the DMSO control. Percent inhibition was calculated using the formula:

% Inhibition = (1 − ((v_Inhibitor_ − B_Inhibitor_)/(v_DMSO_ − B_DMSO_))) × 100

where **v** represents the enzymatic rate and **B** the background (non-enzymatic) rate of substrate hydrolysis. Dose–response curves were generated across multiple inhibitor concentrations, and IC_50_ values were calculated by nonlinear regression using GraphPad Prism (version 10).

Ligand efficiency (LE) was calculated using the equation

LE = −RT × ln(IC_50_)/NHA

where **R** is the gas constant (1.987 × 10^−3^ kcal/mol·K), **T** is temperature (298 K), and **NHA** is the number of non-hydrogen atoms [35]. Lipophilic ligand efficiency (LLE) was calculated as LLE = pIC_50_ − cLogP.

### 2.4. Thermal Shift Assay

The thermal stability of hCA III in the presence of test compounds was assessed using a SYPRO Orange-based fluorescence thermal shift assay on a QuantStudio 3 Real-Time PCR System (Applied Biosystems, Thermo Fisher Scientific, Waltham, MA, USA). Each 20 µL reaction contained 300 µg/mL hCA III, SYPRO Orange dye at a 1:500 dilution (from a 5000× stock), and 40 µM of the test compound. Reactions were prepared in 96-well optical PCR plates and subjected to a temperature ramp from 25 °C to 95 °C, increasing at a rate of 0.5 °C per minute. Fluorescence readings were collected at 1 °C intervals. Melting temperatures (Tm) were obtained by fitting fluorescence versus temperature curves to a Boltzmann sigmoidal model using GraphPad Prism (version 10). Changes in Tm relative to the DMSO control were used to evaluate compound-induced stabilisation or destabilisation of hCA III.

### 2.5. Docking Simulation of hCA III Inhibitor Binding

OEDOCKING suite (version 4.3.3.1, OpenEye Scientific Software, Santa Fe, NM, USA) was used for in silico docking of the identified hCA III inhibitors. Before docking experiments, the three-dimensional (3D) structures of the inhibitors were generated using ChemDraw 16.0 and BIOVIA Discovery Studio Visualizer v21.1.0.20298 (Dassault Systèmes BIOVIA, San Diego, CA, USA). All ligands were docked in their ionised form. The 3D coordinates of hCA III (PDB ID: 1Z93; 2.1 Å; [36]) were used for docking, with all crystallographic water molecules removed. The binding site was defined around the His94 residue using the Make Receptor application from the OEDOCKING suite. Inhibitors were docked into the enzyme binding site using FRED 4.3.3.1 default parameters. Top-ranked poses according to the Chemgauss4 docking scoring function were used in the discussion.

### 2.6. Cell Culture and Compound Treatment

HepG2 human hepatocellular carcinoma cells (ATCC HB-8065, ATCC, Manassas, VA, USA) were used to investigate the cellular effects of selected hCA III inhibitors. Cells were cultured in MEM supplemented with 10% FBS, 1% GlutaMAX, and 1% antibiotic-antimycotic solution (MEM, Euroclone, Milan, Italy; FBS, Cytiva, Little Chalfont, UK; GlutaMAX and antibiotic-antimycotic solution, Gibco, Thermo Fisher Scientific, Paisley, UK), and maintained at 37 °C in a 5% CO_2_ humidified incubator. At 70–80% confluency, cells were detached using TrypLE Express (Gibco, Thermo Fisher Scientific, Paisley, UK) and passaged at a 1:3 ratio.

Stock solutions of the test compounds (100 mM in DMSO) were diluted in complete medium to achieve the desired final compound concentrations, while maintaining a final DMSO concentration of 0.1% in all treatments. Treatment conditions included vehicle-only (DMSO) and untreated control groups. Unless otherwise indicated, all experiments were performed in triplicate.

### 2.7. Selection of a Cellular Model Based on CA3 Expression Profiling

To identify a suitable cellular model for evaluating compound-induced effects on *CA3* expression, baseline *CA3* mRNA levels were quantified across a panel of human cell lines. U87, HeLa, PANC1, HepG2, MCF7, A549, MDA-MB-231, CACO-2, HEK293, MeWo, A375, SCaBER, PC-3, and normal bladder fibroblast cells (NFBs) were cultured under standard conditions appropriate for each cell line. At approximately 70–80% confluence, cells were harvested and total RNA was extracted using the RNeasy Mini Kit (Qiagen, Hilden, Germany) according to the manufacturer’s instructions. cDNA was synthesised from 1 µg of total RNA using PrimeScript^TM^ RT Master Mix (Takara, Kusatsu, Shiga, Japan) following the supplier’s protocol. *CA3* expression was quantified by quantitative PCR (qPCR) using gene-specific primers (see Section 2.11) and TB Green Premix Ex Taq II (Takara Bio, Kusatsu, Shiga, Japan) under standard cycling conditions. Cq values were determined using the instrument software, and relative expression levels were calculated using the ΔCq method. For comparative purposes, expression was normalised to the mean Cq value across the cell line panel and expressed as 2^−ΔCq^. This approach enabled comparison of relative *CA3* expression levels across multiple cell types and selection of an appropriate model for downstream cellular assays.

### 2.8. Cell Viability Assessment Using the MTT Assay

Cell viability following compound exposure was evaluated using the 3-(4,5-dimethylthiazol-2-yl)-2,5-diphenyltetrazolium bromide (MTT) assay. HepG2 cells were treated with test compounds for 48 h. After incubation, 10 µL of MTT reagent (ATCC, Manassas, VA, USA) was added to each well, and plates were incubated at 37 °C and 5% CO_2_ for 3 h. Following this, 110 µL of solubilization/stop solution was added, and incubation continued at room temperature (RT; 22 °C) for 30 min. Absorbance was measured at 570 nm using a Cytation 5 multimode plate reader (Agilent BioTek, Santa Clara, CA, USA), and data analysis was conducted using Gen5 (Version 3.18.17) software (Agilent BioTek, Santa Clara, CA, USA).

### 2.9. Assessment of Intracellular ROS

After HepG2 cells were treated with the test compounds (1 µM) for 48 h, reactive oxygen species (ROS) levels were measured using the Total ROS Assay Kit (Invitrogen, Thermo Fisher Scientific, Paisley, UK). A 20 µM working solution of 2′,7′-dichlorodihydrofluorescein diacetate (DCFDA) was prepared by diluting the 500× stock in pre-warmed serum-free MEM medium. After media removal, 50 µL of the ROS staining solution was added to each well, followed by incubation at 37 °C for 1 h. Positive controls received 200 µM tert-butyl hydroperoxide (tBHP) and were incubated for an additional 30 min. Fluorescence intensity was recorded using a Cytation 5 reader with excitation/emission settings of 488/520 nm.

### 2.10. Assessment of Mitochondrial Membrane Potential

After HepG2 cells were treated with the test compounds (1 µM) for 48 h, mitochondrial membrane potential (MMP) was assessed using a 2 μM JC-1 dye solution. The dye was added to each well, and the red/green fluorescence ratio was measured after 60 min of incubation at 37 °C in a 5% CO_2_. A 50 μM carbonyl cyanide m-chlorophenylhydrazone (CCCP) solution was used as a positive inducer of mitochondrial membrane depolarisation and added 30 min before the end of incubation. The cells were washed with PBS, and fluorescence was measured using the Cytation 5 plate reader. Green fluorescence (monomers) was detected at excitation/emission wavelengths of 485/528 nm, while red fluorescence (aggregates) was measured at 535/590 nm. The red-to-green fluorescence ratio was calculated to determine mitochondrial polarisation. Imaging and quantitative analysis were performed using Gen5 software.

### 2.11. Quantitative Real-Time PCR

To evaluate gene expression changes following treatment with hCA III inhibitors, HepG2 cells were seeded into 6-well plates at a density of 3.5 × 10^5^ cells per well and treated with 1 µM of each compound for 48 h. Total RNA was isolated using the RNeasy Mini Kit (Qiagen, Hilden, Germany), and RNA concentration and purity were determined using a NanoDrop spectrophotometer (Thermo Fisher Scientific, Waltham, MA, USA). One microgram of RNA was reverse transcribed into cDNA in a 20 µL reaction using a Veriti thermal cycler (Applied Biosystems, Thermo Fisher Scientific, Waltham, MA, USA).

Quantitative real-time PCR was carried out using TB Green Premix Ex Taq II (Takara Bio, Kusatsu, Shiga, Japan) on a Bio-Rad CFX96 system (Bio-Rad Laboratories, Hercules, CA, USA). Each reaction was performed in triplicate using specific primers for hCA III (forward: 5′-GGAAGACCTGCCGAGTTGTA-3′; reverse: 5′-GTGAACCAAATGAAGCTCCGC-3′) and GAPDH as the reference gene (forward: 5′-CCTGTTCGACAGTCAGCCG-3′; reverse: 5′-CGACCAAATCCGTTGACTCC-3′). Cycling conditions included an initial denaturation at 95 °C for 30 s, followed by 40 cycles of 95 °C for 5 s and 60 °C for 30 s. Relative gene expression levels were calculated using the ΔΔCq method, with GAPDH used for normalisation.

### 2.12. Statistical Analysis

All statistical analyses were performed using GraphPad Prism version 10 (GraphPad Software, San Diego, CA, USA). Comparisons between groups were made using one-way analysis of variance or two-way analysis of variance (ANOVA), followed by Bonferroni post hoc tests applied where appropriate. Results were considered statistically significant at *p* ≤ 0.05. Significance levels are indicated as follows: * *p* ≤ 0.05, ** *p* ≤ 0.01, *** *p* ≤ 0.001, **** *p* ≤ 0.0001. All experiments were independently performed in triplicate (*n* = 3).

## 3. Results

To identify fragment-like inhibitors of hCA III, we screened a focused set of 25 substituted nicotinic acid analogues (**2**–**26**). The most active compounds were then prioritised for further evaluation by dose–response analysis, ligand efficiency calculations, and a thermal shift assay to support ligand engagement. In parallel with biochemical hit validation, we assessed *CA3* expression across a panel of human cell lines to identify an appropriate cellular model for downstream studies. Based on this profiling, selected compounds were subsequently tested in HepG2 cells to assess cell viability and examine their effects on mitochondrial membrane potential, oxidative stress, and *CA3* gene expression.

### 3.1. Carboxylate Integrity Is Required for hCA III Inhibition

To confirm the importance of the free carboxylate under our assay conditions, we tested four nicotinic acid analogues in which the acid was replaced with chemically distinct substituents (compounds **2**–**5**). Each compound was evaluated at 1 mM using the esterase-based assay with recombinant hCA III (Table 1). Only the parent compound, nicotinic acid (**1**), showed substantial inhibition (71.3 ± 2.8%), whereas all four modified analogues produced minimal inhibition (<10%), regardless of steric bulk or pyridine N-substitution. These inactive analogues therefore served as negative controls for carboxylate-dependent inhibition in this assay. Quercetin inhibited hCA III by 84.5 ± 2.3% at 66.7 µM and was used as a positive control because flavonoids have previously been reported to inhibit CA III in esterase-based assays using 4-nitrophenyl acetate [34], whereas hCA III is less sensitive to classical sulfonamide inhibitors such as acetazolamide. Overall, these data are consistent with the expected requirement for a freely ionisable carboxylate for productive hCA III engagement and support the use of this assay format for screening the substituted nicotinic acid series.

### 3.2. Substitutions at Positions 2 and 6 Enhance Inhibition of hCA III

To explore how substitutions on the nicotinic acid scaffold influence hCA III inhibition, we evaluated 21 commercially available analogues bearing diverse functional groups at positions 2, 5, and 6 of the pyridine ring. These analogues included halogen, hydroxyl, amino, alkylthio, methoxy, and heterocyclic substituents, allowing a broad comparison of how different substitutions affect inhibition in this assay. All compounds were tested at 1 mM against recombinant hCA III (Table 2). At this concentration, most ring substitutions retained measurable inhibition. Several analogues showed inhibition levels comparable to nicotinic acid (approximately 72–83%), including compounds **6**, **8**–**12**, and **14**–**16**, which carry small to moderate substituents with mild polarity or weak electron-withdrawing character. Small hydrophobic substitutions such as ring methylation (compounds **18**–**19**) also retained activity (approximately 73–80%). Other chemically distinct modifications, including the flexible 2-ethoxyethoxy chain (compound **20**) and aromatic substituents such as furan (**23**) or phenyl (**25**), likewise maintained inhibition comparable to nicotinic acid. A subset of compounds showed higher apparent inhibition, including compound **7** (114%) and compound **17** (108%). In addition, compounds **13**, **22**, **24**, and **26** produced near-complete inhibition (approximately 99–104%) under the primary screening conditions, despite structural diversity in their substituents.

Notably, these compounds are substituted at either position 2 (**7** and **17**) or position 6 (**13**, **22**, **24**, and **26**). These results indicate that positions 2 and 6 can be used for further structural modification in future work. Because single-point screening at high concentration can be influenced by assay interference or nonspecific effects, the most active compounds were selected for follow-up testing under more stringent conditions, including detergent control and reduced concentration.

### 3.3. Lead Fragments Identified Based on Inhibition Potency, Efficiency, and Thermal Stabilisation

**Figure 1:**
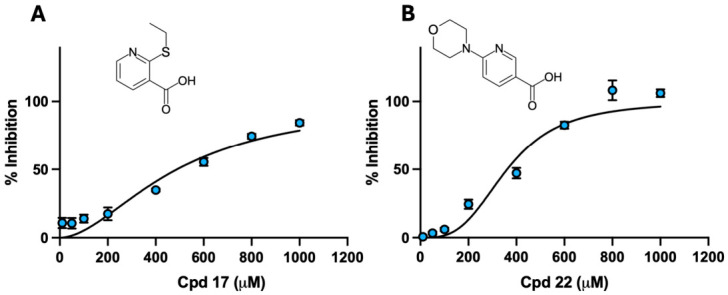
Dose–response curves of the two prioritised hits against hCA III: (**A**) compound **17**; (**B**) compound **22.** Inhibition of hCA III esterase activity was measured using the 4-nitrophenyl acetate assay across a range of inhibitor concentrations. Curves were fitted using a variable-slope four-parameter model in GraphPad Prism. Error bars represent standard deviation from three independent experiments (*n* = 3). IC_50_ values and Hill slopes are reported in Table 3.

Six compounds that showed greater than 85% inhibition at 1 mM were re-tested at 500 µM in the presence of 0.02% Triton X-100 to reduce the risk of nonspecific inhibition. Under these more stringent conditions, only compounds **17** and **22** retained substantial activity (greater than 70%) and were taken forward (Table 3). Dose–response analysis confirmed concentration-dependent inhibition (Figure 1), with IC_50_ values of 487 ± 34 µM for compound **17** (Hill slope 1.8) and 361 ± 16 µM for compound **22** (Hill slope 3.2). Ligand efficiency metrics are summarised in Table 3: compound **17** showed an LE of 0.38 and LLE of 2.13, while compound **22** showed an LE of 0.31 and LLE of 3.60. Thermal shift assays further supported ligand engagement, with positive ΔT_m_ values of 2.3 ± 0.5 °C for compound **17** and 0.61 ± 0.36 °C for compound **22**. Based on this combined detergent control, dose–response, ligand efficiency, and thermal shift profile, compounds **17** and **22** were prioritised for cellular assays and docking studies.

### 3.4. Active Fragment Binding Reveals Specific Interactions with Non-Conserved hCA III Residues

**Figure 2:**
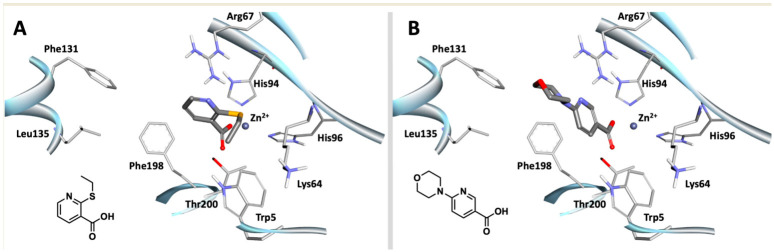
Docking simulation of compounds **17** and **22**. (**A**) Docked poses of compounds **17** and (**B**) **22** in the binding pocket of hCA III (PDB: 1Z93; 2.1 Å). The chemical structure of each docked compound is shown in the lower left corner. Docking was performed using the FRED module of OEDOCKING, as described in Section 2.5.

To explore how the lead fragments might interact with the hCA III active site, molecular docking was performed for compounds **17** and **22** (Figure 2). In the top-ranked poses, both ligands oriented their carboxylate groups toward the catalytic zinc ion in a manner consistent with displacement of the zinc-bound water molecule. For compound **17**, the model suggested a hydrogen bond with Thr200, π–π stacking of the pyridine ring with Phe198, and a polar contact between the pyridine nitrogen and Arg67, while the thioethyl substituent projected toward a hydrophobic region near Lys64 (Figure 2A). For compound **22**, the predicted pose retained zinc coordination and suggested a hydrogen bond with His119, π–π stacking with Phe198, and positioning of the morpholine group near Arg67 toward a pocket involving Phe131, with an additional polar contact to Gln92 (Figure 2B). Overall, these docking poses are consistent with the biochemical requirement for a free carboxylate and suggest how ring substitution may support binding through a combination of polar and hydrophobic interactions within the hCA III pocket, including potential engagement of isoform-variable residues such as Phe198 and Lys64.

### 3.5. CA3 Expression in HepG2 Supports Its Use as a Cellular Model

**Figure 3:**
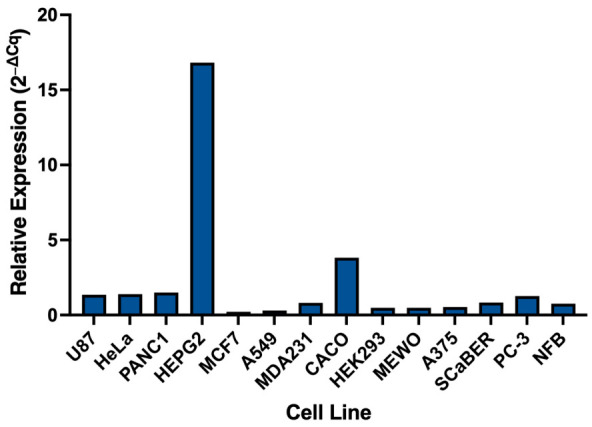
*CA3* expression across a panel of human cell lines. Relative *CA3* mRNA expression levels were determined by qPCR and calculated using the ΔCq method, normalised to the mean Cq value across all cell lines and expressed as 2^−ΔCq^. HepG2 cells showed the highest *CA3* expression (16.8), followed by CACO-2 (3.8).

To identify a suitable cellular system for evaluating compound-induced modulation of *CA3*, we quantified baseline *CA3* mRNA expression across a panel of human cell lines using qPCR (Figure 3).

Expression levels varied across the panel, with most cell lines showing low to moderate expression. HepG2 cells exhibited the highest *CA3* expression, with a relative expression value of 16.8, markedly exceeding all other cell lines tested. In contrast, the majority of cell lines, including U87, HeLa, PANC1, and PC-3, showed expression values close to baseline (approximately 1.2–1.5), while several lines such as MCF7, A549, HEK293, and MeWo displayed low expression levels (≤0.5). CACO-2 cells showed intermediate expression (3.8) but remained substantially lower than HepG2.

These findings demonstrate that *CA3* expression is highly variable across cell types and identify HepG2 cells as a suitable model with sufficiently high baseline expression to enable detection of compound-induced changes. Based on this expression profile, HepG2 cells were selected for subsequent cellular assays.

### 3.6. Compounds ***17*** and ***22*** Differentially Affect CA3 Expression and Cellular Stress Responses in HepG2 Cells

**Figure 4:**
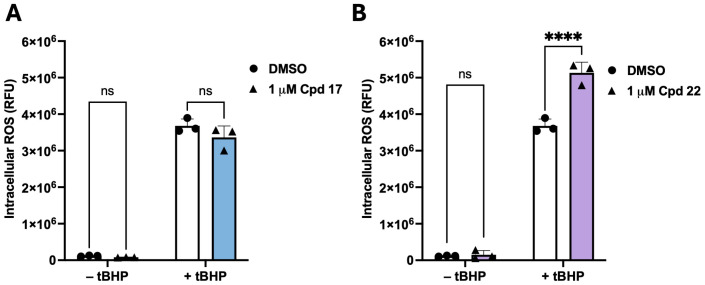

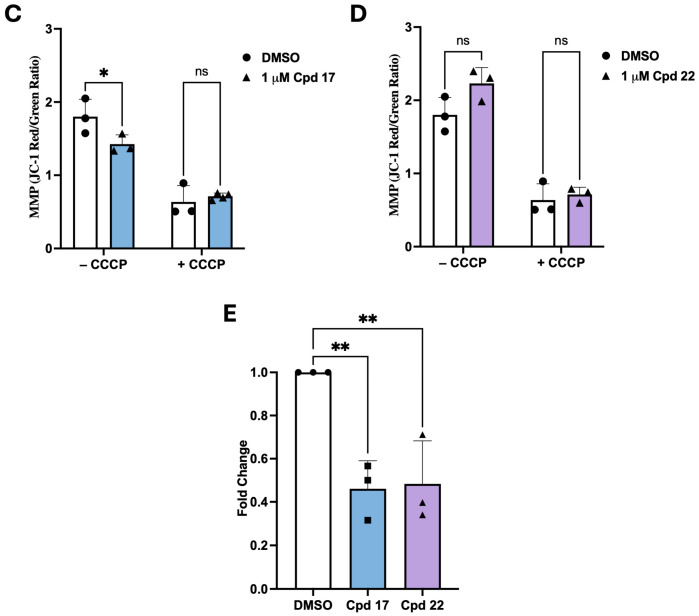
Cellular effects of compounds **17** and **22** in HepG2 cells. (**A**,**B**) Intracellular reactive oxygen species (ROS) levels measured by relative fluorescence units (RFU; higher values indicate greater ROS) following 48 h treatment with compound **17** (**A**) or compound **22** (**B**) at 1 µM, under basal conditions (without tBHP) and oxidative stress conditions (with tBHP). Neither compound significantly altered basal ROS levels. Under oxidative stress, compound **22** significantly increased ROS fluorescence relative to DMSO vehicle (**** *p* ≤ 0.0001), whereas compound **17** produced no significant effect under either condition. (**C**,**D**) Mitochondrial membrane potential (MMP) assessed by the JC-1 red/green fluorescence ratio following 48 h treatment with compound **17** (**C**) or compound **22** (**D**) at 1 µM, in the absence or presence of the mitochondrial uncoupler CCCP. A higher ratio indicates greater mitochondrial polarisation. Compound **17** significantly reduced the red/green ratio in the absence of CCCP (* *p* ≤ 0.05), indicating mitochondrial depolarisation. Neither compound produced a significant effect in CCCP-pretreated cells, consistent with maximal depolarisation already induced by CCCP. Compound **22** did not significantly alter MMP under any condition. (**E**) Relative *CA3* mRNA expression following 48 h treatment with compound **17** or compound **22** at 1 µM, determined by real-time qPCR and normalised to GAPDH using the ΔΔCq method, expressed as fold change relative to DMSO vehicle control. Both compounds significantly downregulated *CA3* mRNA expression compared to DMSO vehicle control (** *p* ≤ 0.01), with fold changes of approximately 0.46 and 0.49 for compounds **17** and **22** respectively, corresponding to approximately 54% and 51% reductions in transcript levels. Data are presented as mean ± SD from three independent biological experiments (*n* = 3); individual data points represent independent biological replicates. Statistical significance was determined using two-way ANOVA (**A**–**D**) or one-way ANOVA (**E**), followed by Bonferroni post hoc correction. (* *p* ≤ 0.05, ** *p* ≤ 0.01, **** *p* ≤ 0.0001). ns, not significant; Cpd, compound; DMSO, dimethyl sulfoxide vehicle control; tBHP, tert-butyl hydroperoxide; CCCP, carbonyl cyanide m-chlorophenylhydrazone.

To determine whether the prioritised hits were associated with measurable cellular phenotypes, compounds **17** and **22** were evaluated in HepG2 cells using a series of functional assays (Figure 4 and  Figure S3). Both compounds were well tolerated across the tested concentration range. Compound **17** showed a modest concentration-dependent increase in MTT absorbance at 10 and 100 µM (*p* < 0.05), which may reflect assay interference rather than a true increase in cell proliferation (Figure S3). Compound **22** produced no significant change in MTT absorbance at any concentration tested (Figure S3). Under oxidative stress conditions induced by tBHP, compound **22** significantly increased intracellular ROS levels compared to the DMSO vehicle (*p* ≤ 0.0001), while neither compound altered basal ROS in the absence of tBHP (Figure 4A,B). Assessment of MMP showed that compound **17** significantly reduced the JC-1 red/green ratio in the absence of CCCP (*p* < 0.05), indicating mitochondrial depolarisation, whereas compound **22** did not significantly alter MMP under any condition tested (Figure 4C,D). Neither compound produced an additional effect in CCCP-pretreated cells, consistent with marked depolarisation already induced by the uncoupler. Both compounds significantly downregulated *CA3* mRNA expression after 48 h of treatment at 1 µM (*p* < 0.01), reducing transcript levels by approximately 54% and 51% for compounds **17** and **22**, respectively (Figure 4E). These findings indicate that compounds **17** and **22** produce distinct cellular responses in HepG2 cells despite their similar biochemical prioritisation.

## 4. Discussion

The lack of suitable chemical tools to probe hCA III function has limited understanding of this isoform in cellular and physiological contexts. Despite its high expression in metabolically active tissues, hCA III remains one of the least explored members of the CA family. Its unusually low catalytic activity, distinct biochemical profile, and the limited availability of well-validated isoform-selective inhibitors have hindered both mechanistic studies and therapeutic exploration. Conventional CA inhibitor discovery approaches, particularly CO_2_ hydration-based assays and sulfonamide-centric campaigns, are often poorly suited to catalytically weak isoforms such as hCA III and have historically provided limited insight into its biological function. In this context, esterase inhibition was used as a biochemical readout of ligand-sensitive perturbation of hCA III, while thermal shift analysis provided orthogonal support for compound-protein interaction. The present study therefore provides an initial chemical starting point for examining whether ligand-dependent perturbation of hCA III is associated with changes in *CA3* expression in human cells, although the mechanistic basis of this relationship remains to be established. More broadly, these findings provide a foundation for developing selective fragment-based tools to investigate hCA III biology, including its proposed roles in redox regulation.

Compared with well-characterised isoforms such as hCA II, the active site of hCA III exhibits distinct structural features that shape its ligand-binding landscape and catalytic profile. Most notably, hCA III contains Lys64 in place of the canonical His64, which is associated with less efficient proton transfer and reduced catalytic turnover. At the pocket entrance, Arg67 introduces additional steric bulk and positive charge relative to the Thr or Ser residues found in several other isoforms. Deeper in the cavity, Phe198 replaces Leu198 in hCA II, narrowing the conical pocket and likely disfavouring bulkier ligands, including many classical sulfonamides. These changes, together with a hydrophobic cluster involving Phe131, Phe198, and Leu197, contribute to a more sterically restricted binding site. Taken together, these features are consistent with a pocket that may favour smaller scaffolds able to accommodate both steric and electrostatic constraints. In light of this architecture, we selected nicotinic acid and related analogues as tractable, fragment-like candidates for probing non-sulfonamide binding modes in hCA III.

Analogues lacking a free carboxylate (**2**–**5**) were inactive and served as negative controls, so subsequent screening focused on ring-substituted nicotinic acids retaining the acid group. Several analogues bearing polar, alkyl, alkylthio, aromatic, or heterocyclic substituents at positions 2 and 6 displayed inhibition comparable to, or greater than, nicotinic acid in the initial assay, indicating that substitution at these positions can be accommodated without loss of apparent activity. This primary screen identified six highly active compounds (**7**, **13**, **17**, **22**, **24**, and **26**). Notably, substitution at position 6 is consistent with prior reports of acipimox binding to hCA III (Ki = 5.4 µM, stopped-flow CO_2_ hydration assay), supporting this region as permissive for ligand engagement [30]. To distinguish specific inhibitors from potential assay artefacts, these highly active compounds were retested in the presence of detergent. Only compounds **17** and **22** retained substantial activity under these conditions, whereas compounds **7**, **13**, **24**, and **26** lost inhibition, suggesting nonspecific or aggregation-based effects. Thermal shift analysis was then used as an orthogonal measure of ligand engagement. Fragment ligands typically produce modest stabilisation of their targets, often within 1–2 °C [37]. Consistent with this expectation, compound **17** increased the hCA III melting temperature by 2.3 ± 0.5 °C, and compound **22** produced a reproducible shift of 0.61 ± 0.36 °C. Given that stabilising interactions are generally associated with specific binding, compounds **17** and **22** were prioritised for further characterisation.

Compounds **17** and **22** showed distinct strengths that supported their prioritisation as lead fragments. As expected for fragment-like hits, both compounds showed sub-millimolar potency and favourable ligand efficiency metrics consistent with their size and polarity. Compound **17** achieved higher ligand efficiency, whereas compound **22** balanced potency and lipophilicity more effectively. Together, these features make them suitable starting points for further chemical elaboration, including fragment growth into solvent-exposed regions. Docking studies provided additional insight into plausible binding modes, with both compounds predicted to coordinate the catalytic zinc ion via the carboxylate and form a combination of polar and hydrophobic contacts within the hCA III pocket. Predicted interactions with residues such as Phe198, Arg67, Lys64, and Gln92 suggest opportunities to engage isoform-variable features of the active site. These docking poses are also consistent with earlier molecular modelling studies on bovine CA III, which suggested related binding orientations for nicotinic acid analogues in the 1Z93-based pocket [33,38]. These structural and biochemical observations prompted us to examine whether these hits were also associated with changes in oxidative stress markers and *CA3* expression in HepG2 cells.

In HepG2 cells, compounds **17** and **22** showed divergent cellular behaviour despite comparable biochemical prioritisation, indicating that progression from biochemical activity to cellular phenotype is not straightforward for hCA III-directed fragments. Neither compound showed evidence of overt cytotoxicity in the MTT assay, although the increased MTT signal observed with compound **17** at higher concentrations was not interpreted as enhanced proliferation and may instead reflect altered reductive activity or assay interference. More informative differences emerged in the stress-related readouts, where compound **22** increased ROS under oxidative stress, while compound **17** disrupted MMP in the absence of CCCP pretreatment. Notably, both compounds downregulated *CA3* mRNA after 48 h, and this shared transcriptional effect, despite otherwise distinct cellular profiles, suggests that *CA3* downregulation may represent a useful readout for future mechanistic studies. However, the cellular assays were performed at 1 µM, a concentration chosen to minimise nonspecific cellular effects and well below the IC_50_ values measured in the surrogate esterase assay, so the present data do not establish that these phenotypes arise from direct hCA III inhibition in cells. Instead, they identify associated cellular responses of prioritised hCA III-interacting fragment hits and highlight the importance of combining biochemical, biophysical, and cellular readouts when studying catalytically weak isoforms such as hCA III. Taken together, these findings support both compounds **17** and **22** as fragment-like starting points worthy of further exploration, with their divergent cellular behaviours providing complementary opportunities for mechanistic follow-up and chemical optimisation.

This mechanistic complexity is also relevant when the present findings are considered alongside previous reports linking CA III expression to oxidative-stress protection. Previous work showed that overexpression of CA III protected cells against hydrogen peroxide-induced oxidative stress [15]. However, that study used rat CA III cDNA transfected into NIH/3T3 mouse fibroblasts [15], whereas the present work examined pharmacological perturbation of endogenous human *CA3* in HepG2 cells, which showed the highest baseline *CA3* expression in our cell-line panel. Direct comparison between these systems therefore requires caution. They differ in species, cell type, and baseline *CA3* context, and CA III has also been reported to vary across species in sequence, tissue distribution, and biochemical properties [11,39,40]. In addition, our study measured *CA3* at the mRNA level after 48 h and did not determine the corresponding change in hCA III protein abundance. The observed transcriptional decrease therefore does not necessarily imply a proportional reduction in protein function over the same timescale. The concurrent downregulation of *CA3* by both compounds is therefore unlikely, by itself, to explain the distinct ROS and mitochondrial phenotypes observed here and may instead represent a downstream or compensatory response to altered cellular state. In this context, the divergent behaviours of compounds **17** and **22** suggest that multiple mechanisms contribute to the cellular readouts and that the sequence linking hCA III perturbation, *CA3* expression, oxidative stress, and mitochondrial effects remains to be established experimentally.

Beyond this unresolved mechanistic picture, several aspects of hCA III modulation remain to be clarified through complementary studies. Further development of compounds **17** and **22** will require fragment-based optimisation, including fragment growth, merging, or linking, to improve affinity while maintaining ligand efficiency. Inhibitor activity was assessed primarily using an esterase-based assay rather than the classical CO_2_ hydration readout; because hCA III is catalytically weak for CO_2_ hydration, different assay formats may capture different aspects of enzyme behaviour and influence apparent potency. Although prior mechanistic studies suggest that, in CA III, hydrolysis of 4-nitrophenyl acetate may reflect a mode of interaction distinct from that responsible for CO_2_ hydration, the physiological significance of this esterase activity remains unresolved. The IC_50_ values obtained with 4-nitrophenyl acetate are therefore best viewed as assay-specific measures of activity rather than direct proxies for potency in cellular systems. Importantly, the orthogonal thermal shift assay was performed at 40 µM, substantially below the IC_50_ values measured in the esterase assay, showing that compound-protein interaction with purified hCA III could be detected under conditions below those required for half-maximal inhibition of hydrolysis of the surrogate substrate. The present assay should therefore be regarded as a practical biochemical screen for ligand-sensitive perturbation of hCA III rather than a direct measure of its proposed non-catalytic biological functions. Future work should benchmark these compounds across additional CA activity assays and extend profiling to other CA isoforms, as selectivity was not assessed here. In addition, the downregulation of *CA3* expression observed in HepG2 cells raises mechanistic questions that remain unresolved, including whether this transcriptional response is a direct consequence of hCA III engagement or a secondary cellular effect. Structural validation of binding mode also remains an important unmet objective. Extensive co-crystallisation attempts with both compounds using recombinant hCA III were unsuccessful in our laboratory, consistent with the broader difficulty of obtaining ligand-bound structures of this isoform. Solution-phase approaches such as saturation transfer difference NMR and hydrogen-deuterium exchange mass spectrometry may provide valuable alternatives for characterising fragment engagement and represent priority directions for future work.

This work offers a practical starting point for studying hCA III with non-sulfonamide chemistry. Across a focused set of nicotinic acid derivatives, we identified fragment-like hits that remained active under detergent control, showed orthogonal support by thermal shift, and produced measurable cellular readouts, including reduced *CA3* expression in HepG2 cells. Together, these results suggest that nicotinic acid-derived fragments can serve as tractable chemical tools for probing hCA III biology beyond classical CO_2_ hydration assays. These findings also provide a useful starting point for future studies aimed at clarifying the non-catalytic functions of hCA III in redox-linked cellular contexts. More broadly, our workflow illustrates that catalytically weak isoforms can still be interrogated using alternative assay formats combined with orthogonal validation and cell-based testing. While the mechanism linking small-molecule engagement to changes in *CA3* transcription remains to be defined, this observation provides a useful cellular readout for future studies aimed at clarifying the physiological roles of hCA III in redox-linked contexts and metabolic stress.

## 5. Conclusions

This study advances the development of chemical tools for hCA III by identifying fragment-like, non-sulfonamide nicotinic acid derivatives with reproducible biochemical activity and measurable cellular readouts. Using an esterase-based assay with detergent control, orthogonal thermal shift validation, and follow-up cellular testing in HepG2 cells, we prioritised compounds **17** and **22** as lead fragments. Both compounds inhibited recombinant hCA III and were associated with reduced *CA3* mRNA expression after treatment, while their cellular phenotypes diverged, with compound **22** altering ROS under oxidative stress conditions and compound **17** affecting mitochondrial membrane potential. Although selectivity across CA isoforms and the mechanism linking enzymatic engagement to transcriptional changes were not addressed here, these findings provide a practical starting point for fragment-based optimisation and for probing hCA III biology in redox-linked cellular contexts. Further studies are needed to define the mechanisms underlying the distinct cellular behaviours of compounds **17** and **22**, including their intracellular target engagement, selectivity across other CA isoforms, particularly mitochondrial isoforms, and possible off-target mitochondrial effects, alongside continued fragment optimisation toward more potent leads.

## Figures and Tables

**Table 1:** Inhibition of hCA III by nicotinic acid and carboxyl-modified analogues.

	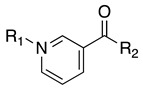		
Compound	R_1_	R_2_	Inhibition (%) at 1000 µM
**1**		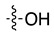	71.3 ± 2.8
**2**		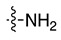	2.7 ± 6.7
**3**	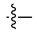	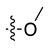	8.9 ± 1.1
**4**		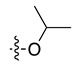	5.2 ± 5.6
**5**		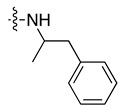	7.7 ± 3.2
Quercetin *	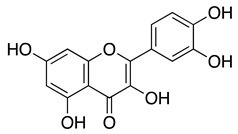		84.5 ± 2.3

* Quercetin was used as a positive control and tested at 66.7 µM. Data are presented as mean ± SD (*n* = 3).

**Table 2:** Inhibition of hCA III by nicotinic acid derivatives substituted on the pyridine ring.

	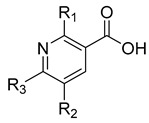			Inhibition (%)
Compound	R_1_	R_2_	R_3_	1000 µM
**6**	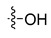			72.5 ± 3.2
**7**	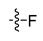			114.0 ± 8.3
**8**	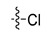			79.4 ± 1.3
**9**	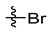			79.5 ± 3.0
**10**	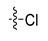	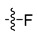	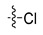	82.7 ± 7.2
**11**	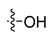	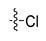		72.8 ± 9.3
**12**	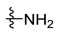	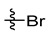		75.4 ± 2.2
**13**			** 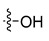 **	99.8 ± 1.4
**14**	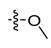			75.0 ± 6.3
**15**	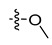		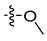	77.2 ± 1.0
**16**	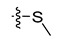			72.0 ± 4.2
**17**	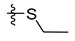			108.1 ± 4.6
**18**	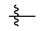		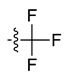	72.9 ± 6.4
**19**			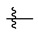	73.6 ± 5.9
**20**			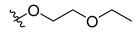	79.5 ± 8.4
**21**			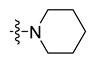	79.5 ± 5.0
**22**			** 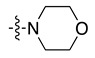 **	104.0 ± 13.0
**23**			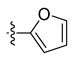	83.0 ± 1.0
**24**			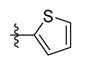	99.8 ± 8.5
**25**			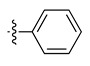	65.7 ± 5.8
**26**			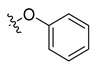	100.3 ± 10.0

**Table 3:** Comparative fragment properties of identified hits. ^a^

	17	22
MW	183.2	208.2
NHA	12	15
cLogP	1.176	−0.156
IC_50_ (×10^−6^ M)	487 ± 34	361 ± 16
Hill Slope	1.8	3.2
pIC_50_	3.31	3.44
LE	0.38	0.31
LLE	2.13	3.60
ΔT_m_ (°C)	2.3 ± 0.5	0.61 ± 0.36
% Inhibition (500 µM + 0.02% Triton)	71.0 ± 7.3	94.5 ± 1.0

^a^ Abbreviations: MW, molecular weight; NHA, non-hydrogen atom count; cLogP, calculated octanol/water partition coefficient; LE, ligand efficiency; LLE, lipophilic ligand efficiency; ΔTm, change in melting temperature. cLogP values were calculated using ChemDraw Professional 16.0 Chemical Properties.

## Data Availability

The datasets used and/or analysed during the current study are available from the corresponding author on reasonable request.

## Supplementary Materials

The following supporting information can be downloaded at: https://www.mdpi.com/article/10.3390/biom16040599/s1, Figure S1: SDS–PAGE (12%) analysis of hCA III purification by immobilized metal affinity chromatography (IMAC) using Ni^2+^-NTA resin. Figure S2. Size-exclusion chromatography (SEC) profile of purified hCA III. Figure S3. Effect of compounds **17** and **22** on HepG2 cell viability assessed by MTT assay.
