# Supplementary material for: Fragment-Derived Nicotinic Acid Analogues Inhibit hCA III and Downregulate CA3 Expression in HepG2 Cells
Source: Biomolecules. 2026 Apr 17;16(4):599. doi: 10.3390/biom16040599 (PMC13113631; doi:10.3390/biom16040599)
Supplement: Supplementary file 1 [file biomolecules-16-00599-s001.zip › biomolecules-4194297-supplementary.pdf]

Fragment-Derived Nicotinic Acid Analogues Inhibit hCA III and Downregulate *CA3* Expression in HepG2 Cells

[1] Department of Pharmaceutical Sciences, School of Pharmacy, The University of Jordan, Amman, Jordan.
[2] Cell Therapy Center, The University of Jordan, Amman, Jordan.
[3] School of Biochemistry, University of Bristol, Bristol, United Kingdom.
[4] Bristol Synthetic Biology Centre BrisSynBio, Bristol, United Kingdom.
[5] Max Planck Bristol Centre for Minimal Biology, Bristol, United Kingdom.
[6] School of Chemistry, University of Bristol, Bristol, United Kingdom.
[7] Department of Pharmacology, University of Oxford, Oxford, United Kingdom.
*Author to whom correspondence should be addressed: email a.abuhammad@ju.edu.jo
ORCID ID: 0000-0003-4978-5059

Supplementary Materials

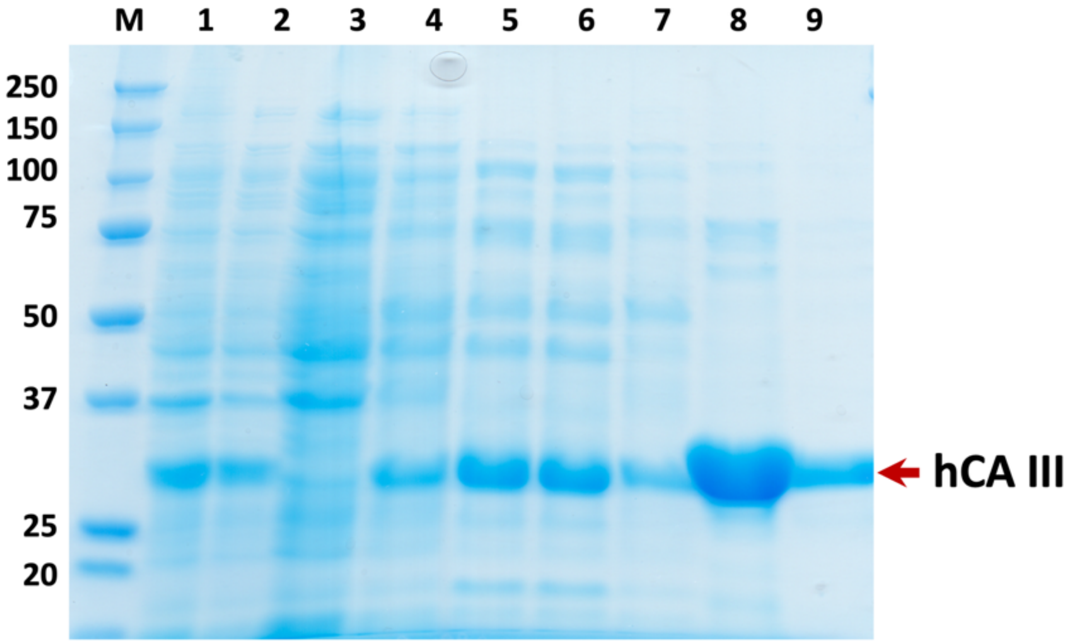

Figure S1. SDS–PAGE (12%) analysis of hCA III purification by immobilized metal affinity chromatography (IMAC) using Ni^{2+}-NTA resin.

Lane M: molecular weight marker (Precision Plus Protein Standards, Bio-Rad). Lane 1: whole-cell lysate. Lane 2: soluble fraction. Lane 3: flow-through. Lanes 4–7: wash fractions with 30 mM imidazole. Lanes 8–9: elution fractions with 250 mM imidazole.

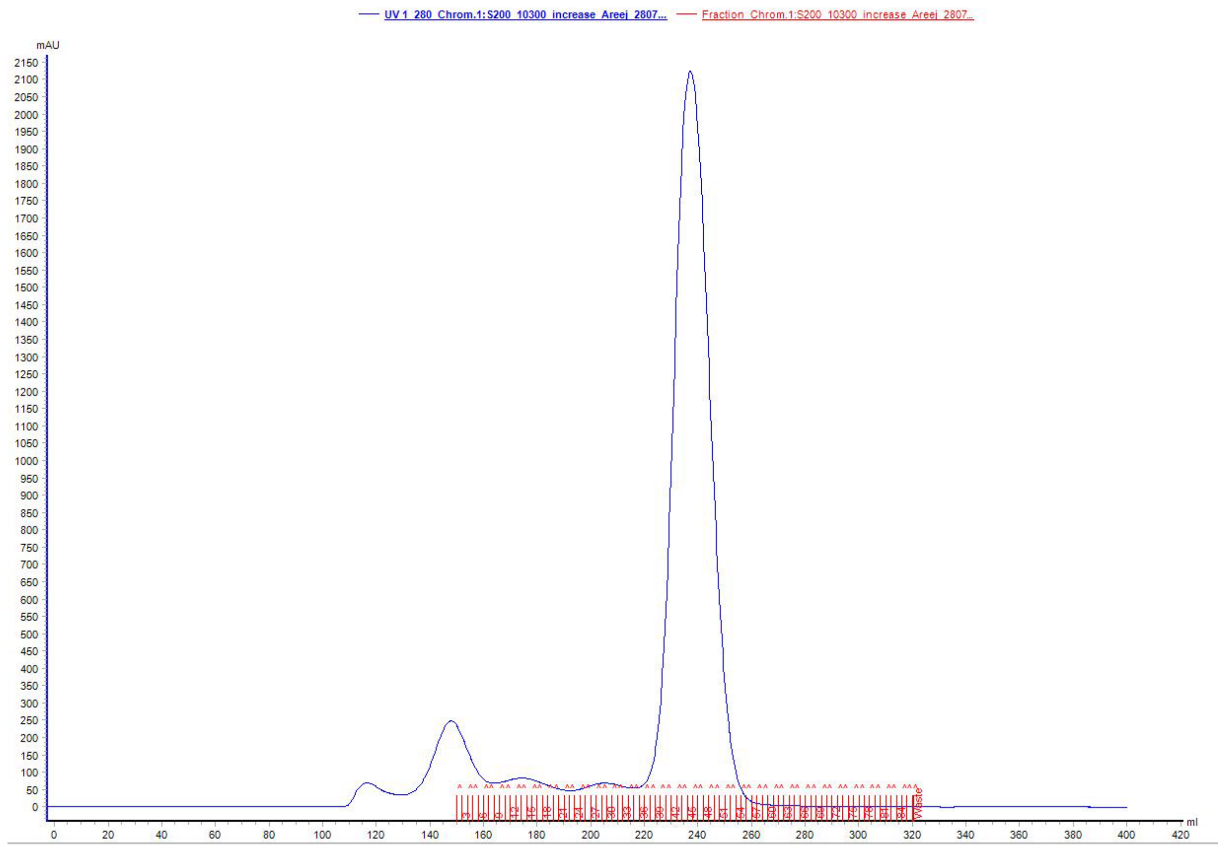

Figure S2. Size-exclusion chromatography (SEC) profile of purified hCA III.

The A280 absorbance trace was recorded during SEC on a Superdex 200 10/300 GL column, showing a single, symmetric peak corresponding to the monomeric form of hCA III. The column was equilibrated and run in 20 mM HEPES (pH 7.5), 150 mM NaCl, 0.5 mM TCEP, 5% glycerol, and fractions corresponding to the main peak were pooled for downstream analyses.

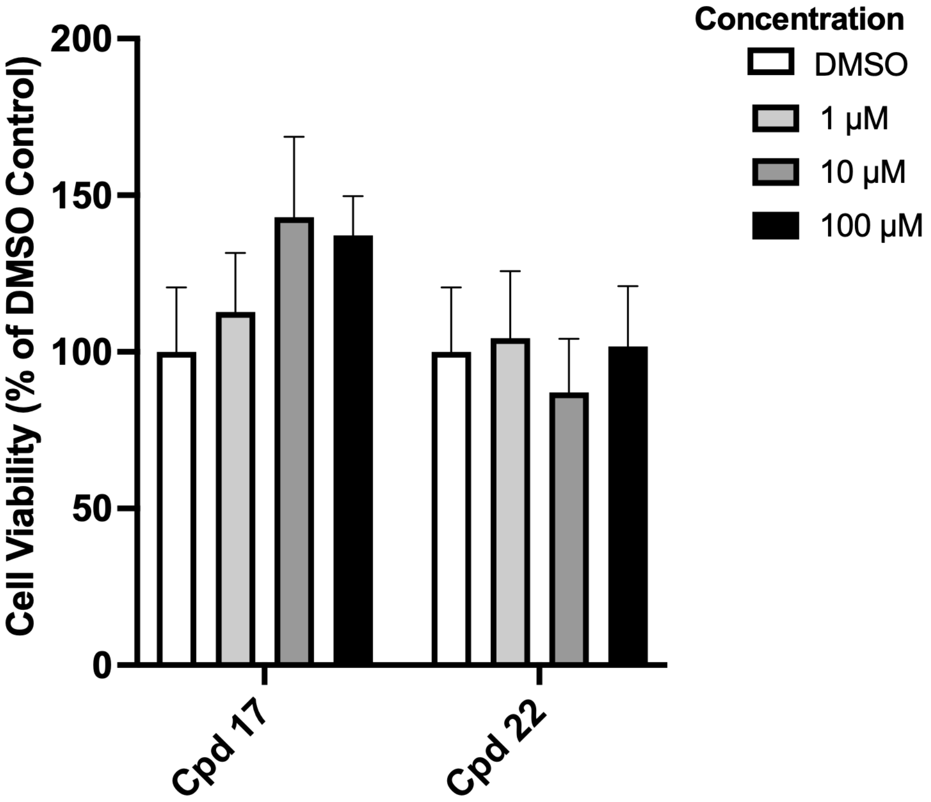

Figure S3. Effect of compounds 17 and 22 on HepG2 cell viability assessed by MTT assay.
Cell viability following 48 h treatment with compounds **17** and **22** at 1, 10, and 100 μM, expressed as percentage of the DMSO vehicle control. Both compounds were well tolerated across the tested concentration range, with no substantial reduction in viability observed at any concentration. The modest increase in MTT signal observed for compound **17** at higher concentrations was not interpreted as enhanced proliferation and may instead reflect altered reductive activity or assay interference. Data are presented as mean ± SD from 3 independent biological experiments (n = 3). Cpd, compound; DMSO, dimethyl sulfoxide vehicle control.
